# Immunofocusing on the conserved fusion peptide of HIV envelope glycoprotein in rhesus macaques

**DOI:** 10.1101/2024.11.27.625755

**Published:** 2024-11-29

**Authors:** Payal P. Pratap, Christopher A. Cottrell, James Quinn, Diane G. Carnathan, Daniel L.V. Bader, Andy S. Tran, Chiamaka A. Enemuo, Julia T. Ngo, Sara T. Richey, Hongmei Gao, Xiaoying Shen, Kelli M. Greene, Jonathan Hurtado, Katarzyna Kaczmarek Michaels, Elana Ben-Akiva, Joel D. Allen, Gabriel Ozorowski, Max Crispin, Bryan Briney, David Montefiori, Guido Silvestri, Darrell J. Irvine, Shane Crotty, Andrew B. Ward

## Abstract

During infection, the fusion peptide (FP) of HIV envelope glycoprotein (Env) serves a central role in viral fusion with the host cell. As such, the FP is highly conserved and therefore an attractive epitope for vaccine design. Here, we describe a vaccination study in non-human primates (NHPs) where glycan deletions were made on soluble HIV Env to increase FP epitope exposure. When delivered via implantable osmotic pumps, this immunogen primed immune responses against the FP, which were then boosted with heterologous trimers resulting in a focused immune response targeting the conserved FP epitope. Although autologous immunizations did not elicit high affinity FP-targeting antibodies, the conserved FP epitope on a heterologous trimer further matured the lower affinity, FP-targeting B cells. This study suggests using epitope conservation strategies on distinct Env trimer immunogens can focus humoral responses on desired neutralizing epitopes and suppress immune-distracting antibody responses against non-neutralizing epitopes.

## Full Text Availability

The license terms selected by the author(s) for this preprint version do not permit archiving in PMC. The full text is available from the preprint server.

