# Supplementary material for: Immunofocusing on the conserved fusion peptide of HIV envelope glycoprotein in rhesus macaques
Source: bioRxiv. 2025 Jun 5:2024.11.27.625755. Originally published 2024 Nov 29. Preprint. [Version 2] doi: 10.1101/2024.11.27.625755 (PMC11623688; doi:10.1101/2024.11.27.625755)
Supplement: Supplement 1 [file media-1.pdf]

Supplemental Data and Figures

Title: Immunofocusing on the conserved fusion peptide of HIV envelope glycoprotein in rhesus macaques

Payal P. Pratap[1,2], Christopher A. Cottrell[2,3], James Quinn[2,4], Diane G. Carnathan[5], Daniel L.V. Bader[1,2,3], Andy S. Tran[1], Chiamaka A. Enemuo[5], Julia T. Ngo[5], Sara T. Richey[1], Hongmei Gao[6], Xiaoying Shen[6], Kelli M. Greene[6], Jonathan Hurtado[2,3], Katarzyna Kaczmarek Michaels[7], Elana Ben-Akiva[7], Ashley Lemnios[3], Mariane B. Melo[2,3,8], Joel D. Allen[9], Gabriel Ozorowski[1,2], Max Crispin[9], Bryan Briney[2,3], David Montefiori[6], Guido Silvestri[5], Darrell J. Irvine[2,7,8,10], Shane Crotty[2,3], and Andrew B. Ward[1,2*]

Author Affiliations

[1] Department of Integrative Structural and Computational Biology, The Scripps Research Institute, La Jolla, CA 92037, USA

[2]Center for HIV/AIDS Vaccine Development (CHAVD), The Scripps Research Institute, La Jolla, CA, 92037, USA

[3] Department of Immunology and Microbiology, The Scripps Research Institute, La Jolla, CA 92037, USA

[4]La Jolla Institute for Immunology, La Jolla, CA 92037, USA

[5]Division of Microbiology and Immunology, Emory National Primate Research Center, Emory University, Atlanta, GA 30329, USA.

[6]Duke Human Vaccine Institute and Department of Surgery, Duke University Medical Center, Durham, NC, USA.

[7]Koch Institute for Integrative Cancer Research, Massachusetts Institute of Technology, Cambridge, Massachusetts 02139, USA

[8]Howard Hughes Medical Institute, 4000 Jones Bridge Rd., Chevy Chase, MD, 20815

[9]School of Biological Sciences, University of Southampton, Southampton, SO17 1BJ, UK

[10]Department of Biological Engineering, Massachusetts Institute of Technology, Cambridge, Massachusetts 02139, USA

[*]Correspondence: andrew@scripps.edu

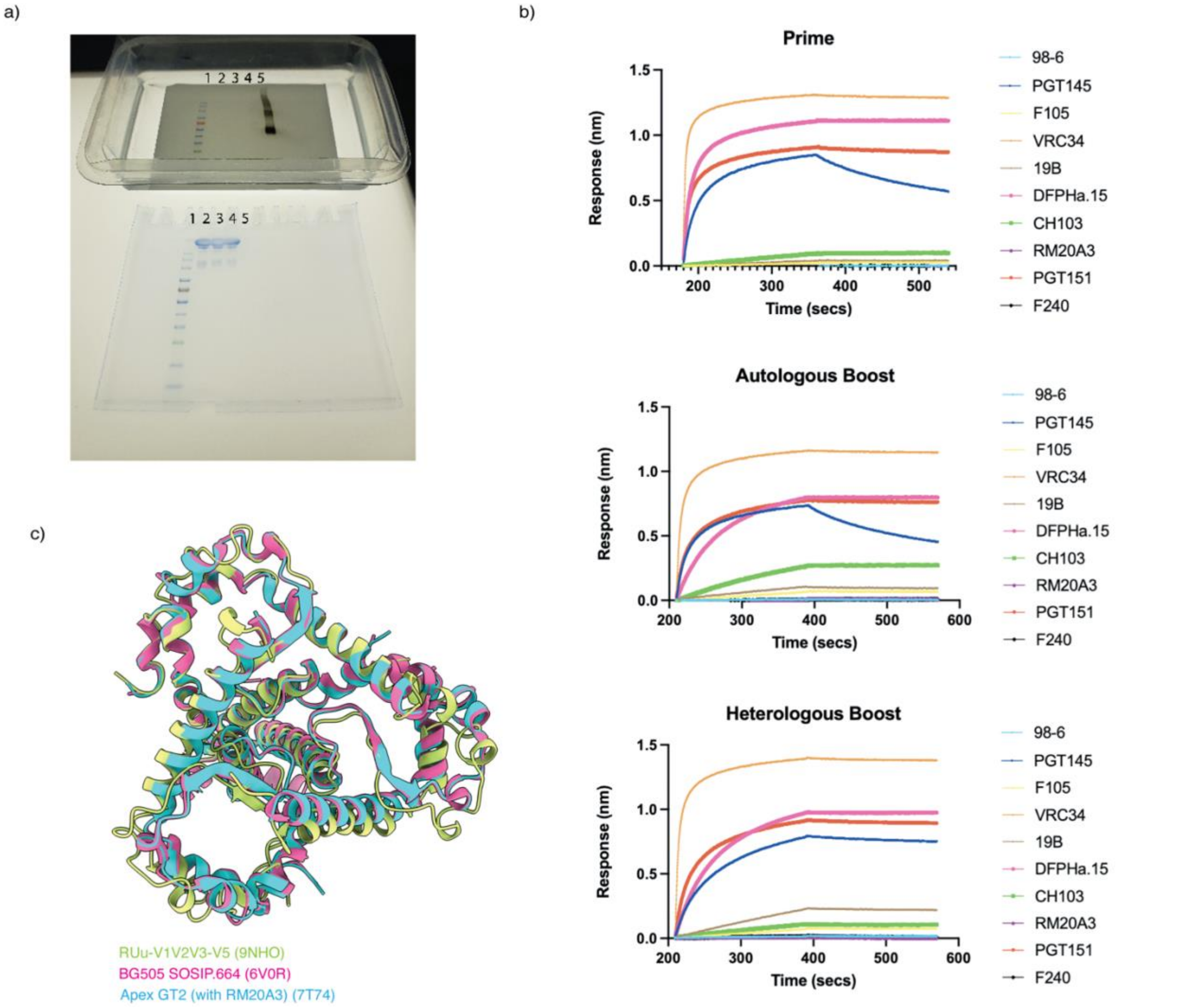

Fig S1. Immunogen Design and Antibody Binding Profiles

a) Anti-Flag Tag Western blot (top panel) and SDS-PAGE gel (bottom panel) of the immunogens with the same, following lanes: (1) Color Pre-stained Protein Standard, Broad Range (10-250 kDa) (NEB #P7719) (2) Prime Immunogen (3) Autologous Boost Immunogen (4) Heterologous Boost Immunogen (5) positive control recombinant Posi-tag epitope tag protein

b) BLI assessment of antigenicity against a panel of anti-Env mAbs: PGT145 (apex), VRC34 (FP), DFPHa.15 (FP), CH103 (CD4bs), RM20A3 (base), PGT151 (FP), F105 (anti-gp120, nnAb), 19b (V3), F240 (anti-gp41), 98-6 (anti-gp41).

c) Bottom view overlap of gp41 subunits of unliganded BG505 SOSIP.664 (501C-605C) in pink, Apex GT2 bound with RM20A3 (501C-605C) in blue and Autologous Boost cryoEMPEM map Uu-V1V2V3-5 (501C-663C) in green.

a)

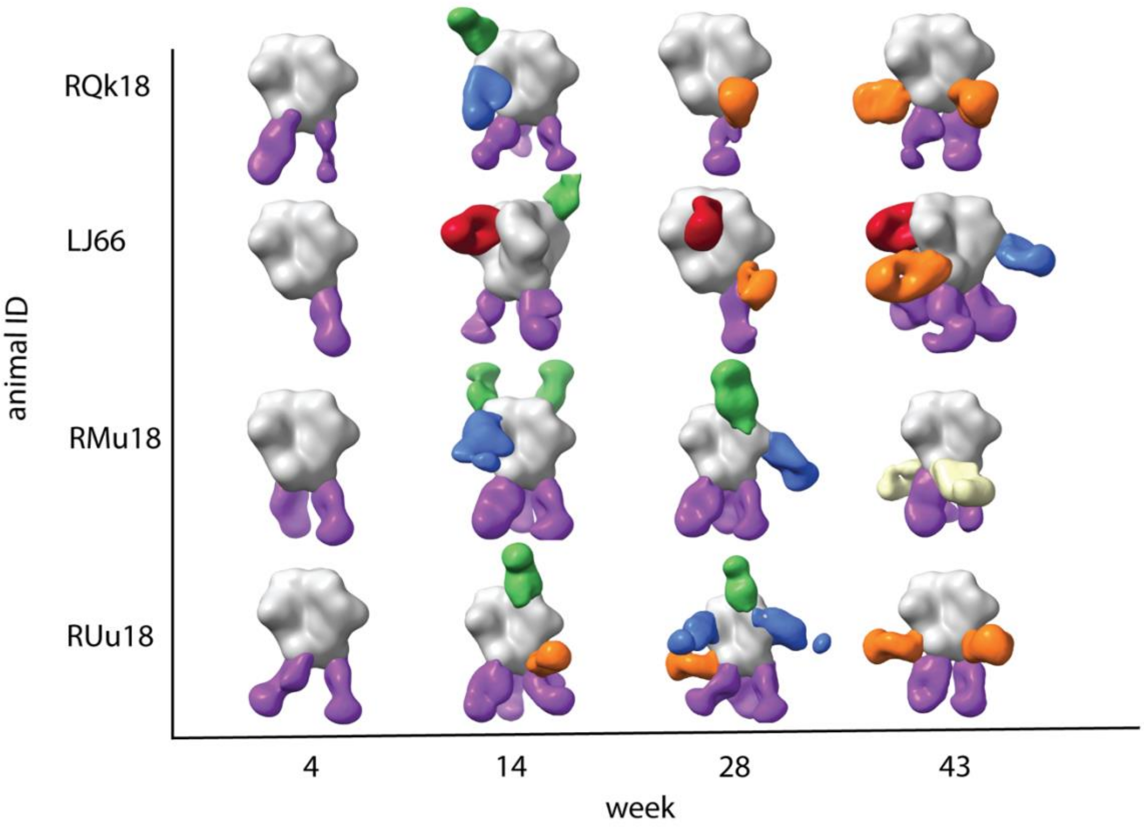

b)

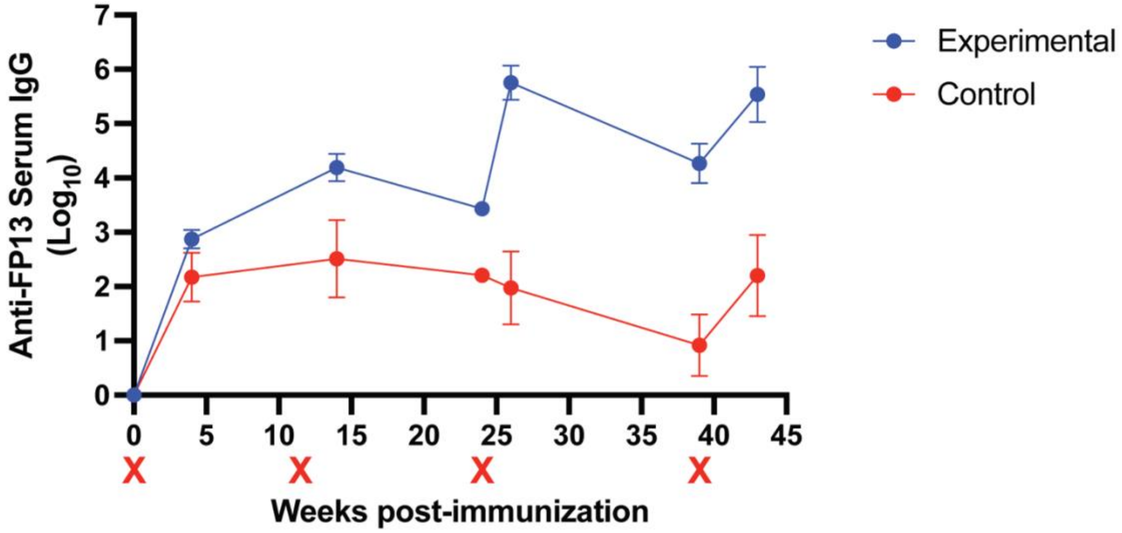

Fig S2. nsEMPEM volumetric models and serum FP-ELISA

a) Representative volumetric models of nsEMPEM analysis of two experimental group (RQk18 and LJ66) and two control group (RMu18 and RUu18) animals
b) Serum FP-ELISA using FP-13 as a probe to detect serum anti-FP levels with experimental group in blue and control group in red.

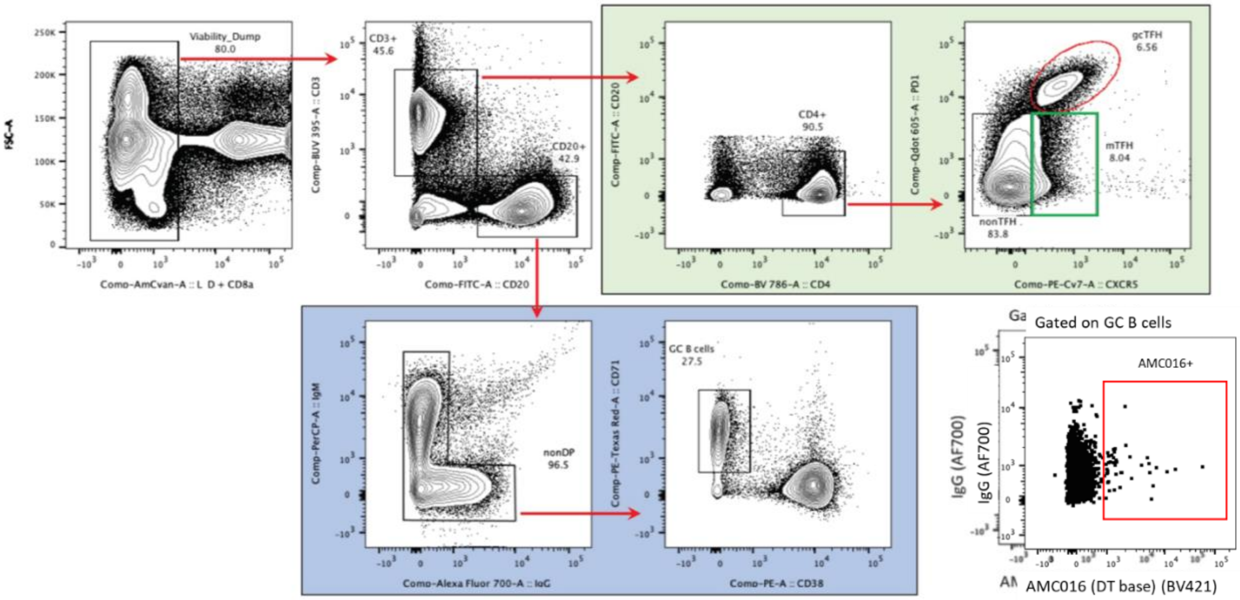

Fig S3. Representative flow plots for FNAs

Representative gating strategy and flow plots used for FNA analysis

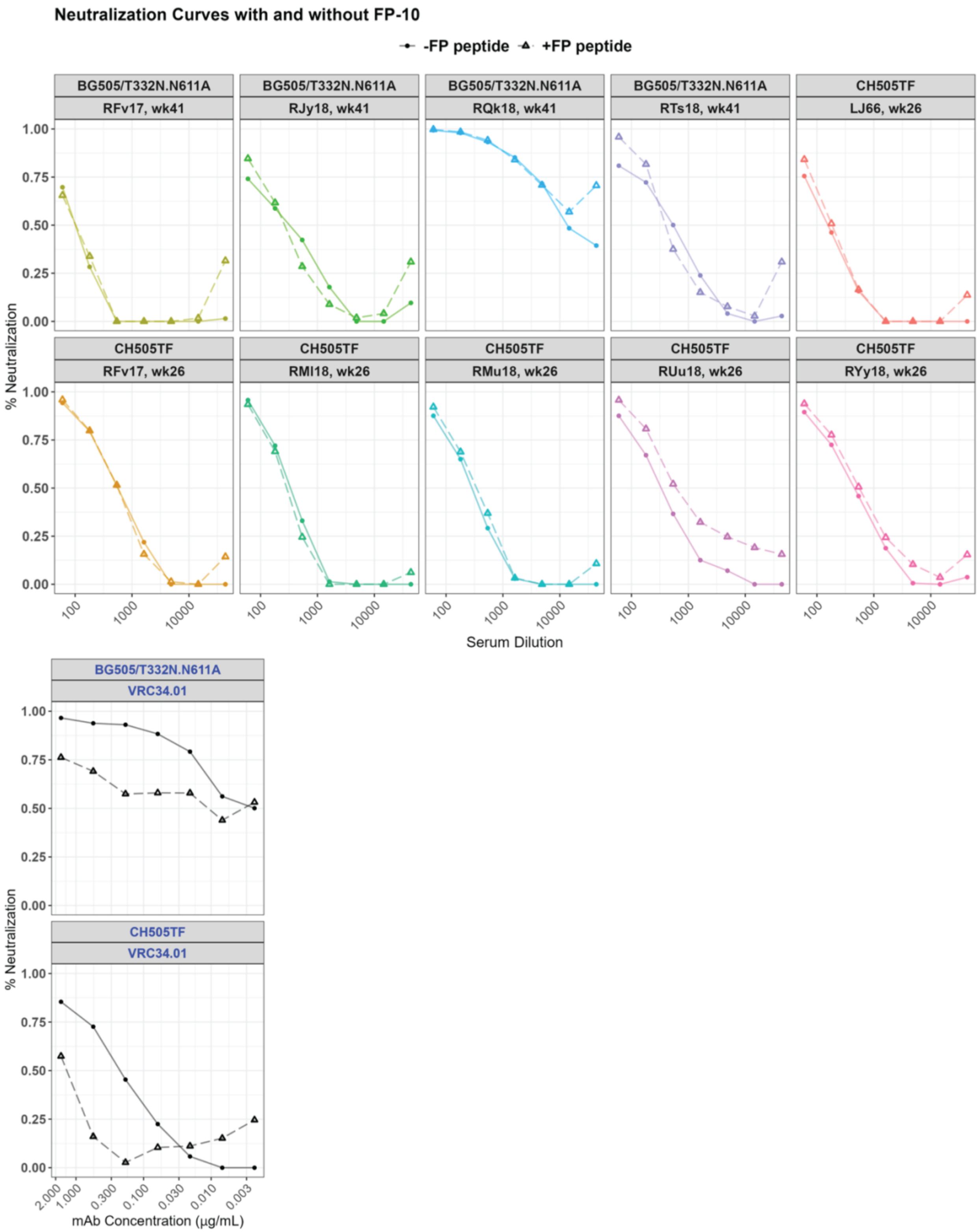

Fig S4. FP Peptide Competition Neutralization Assays

Serum neutralization assays with (dotted-lines) versus without (solid-lined) FP-10 spiked in to capture any FP-targeting antibody responses

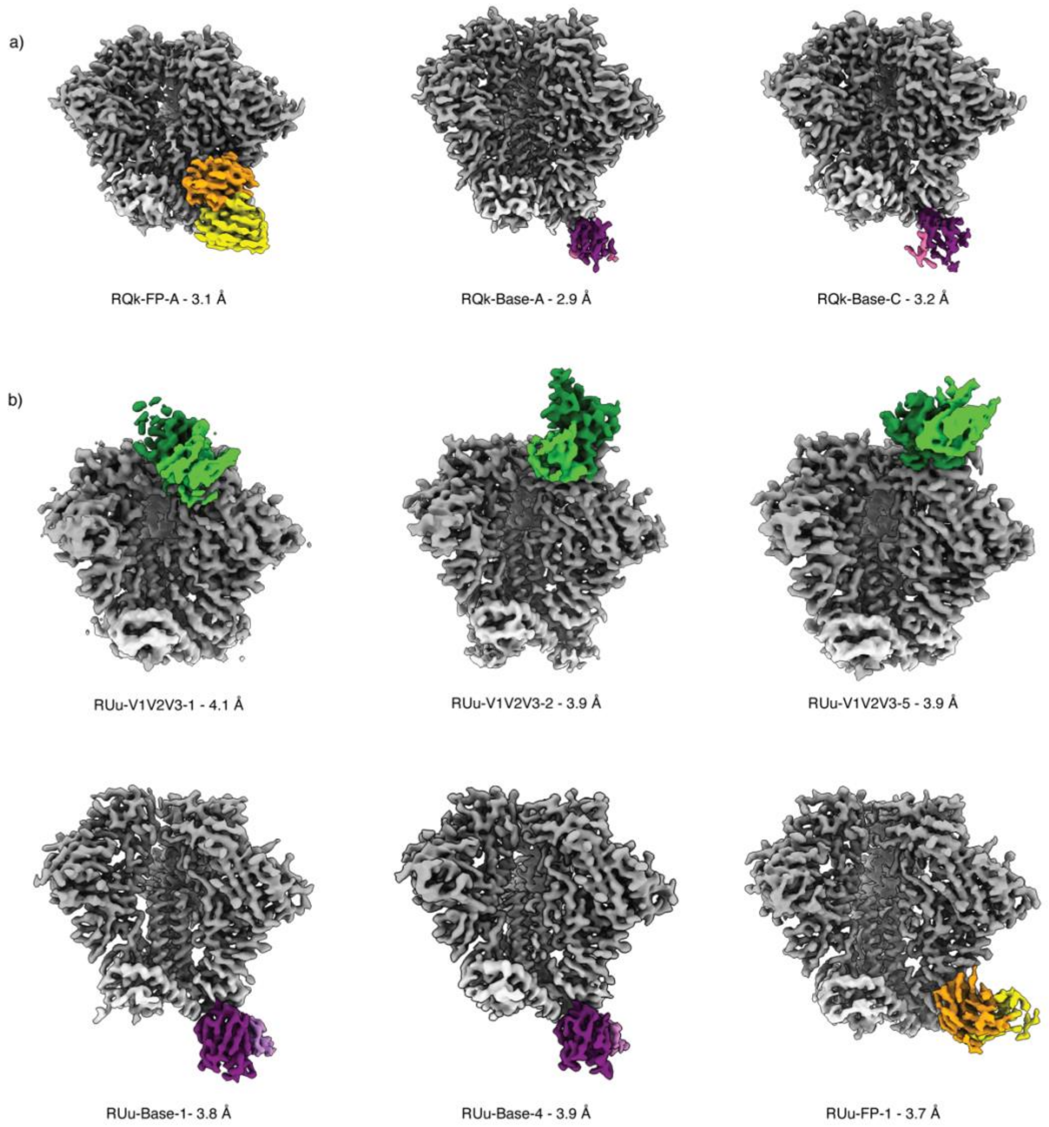

Fig S5. CryoEMPEM High Resolution Maps

a) Three high resolution maps were resolved from animal RQk18 week 43 polyclonal Fab response in complex with Heterologous Boost, including one against the FP and two maps against the base.
b) Six high resolution maps were resolved from animal RUu18 week 14 polyclonal Fab response in complex with Autologous Boost, including three against the V1V2V3 regions, two against the base and one against the C1/C2 non-FP-specific region.

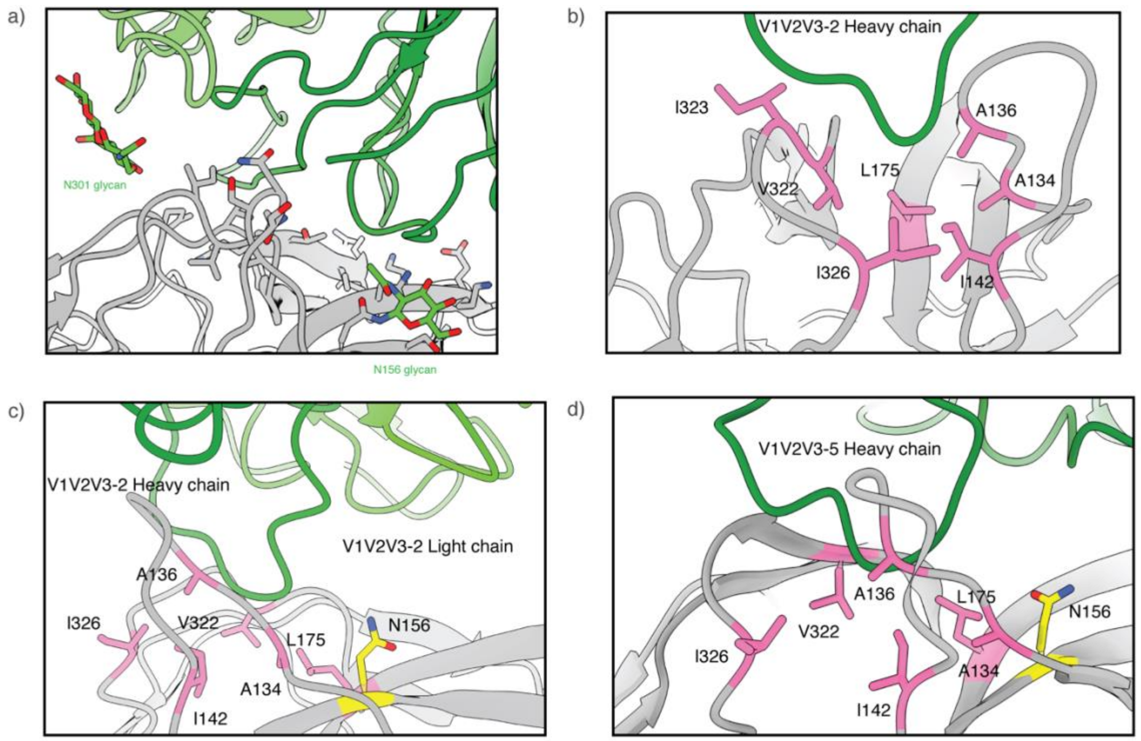

Fig S6. V1V2V3 Off-Target Responses

a) RUu-V1V2V3-1 (green) responses bracketed by N301 and N156 glycans
b) RUu-V1V2V3-2 view (green) 1 shows interactions with hydrophobic patch (pink)
c) RUu-V1V2V3-2 (green) view 2 shows lack of N156 glycan presence (yellow)
d) RUu-V1V2V3-5 (green) shows lack of N156 glycan presence (yellow) as well as hydrophobic patch (pink)

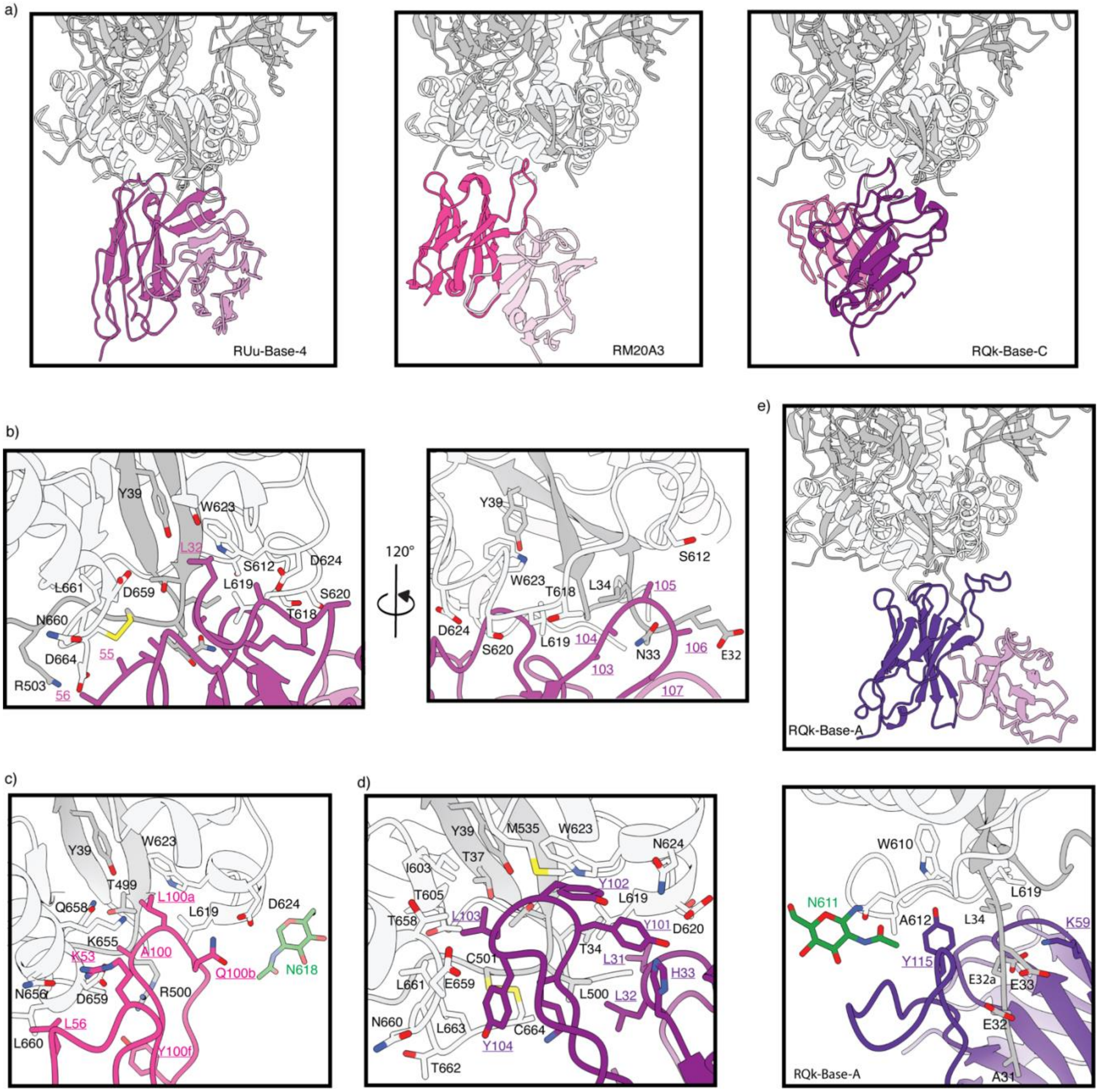

Fig S7. Base Off-target Responses

a) Base Antibodies target the tryptophan clasp (RUu-Base-4 – left panel; RM20A3 (PDB 7T74) – middle panel; RQk-BaseC – right panel)
b) RUu-Base-4 targets the tryptophan clasp with HCDR1 and HCDR2 (left panel) and N-terminus of gp120 with its HCDR3 (right panel)
c) RM20A3 (PDB 7T74) targets the tryptophan clasp with its HCDR3 interactions
d) RQk-Base-C targets tryptophan clasp with its HCDR3 interactions
e) RQk-Base-A targets the N-terminus of gp120 (top panel) with HCDR3 interactions with the charged residues at the N-terminus (bottom panel)

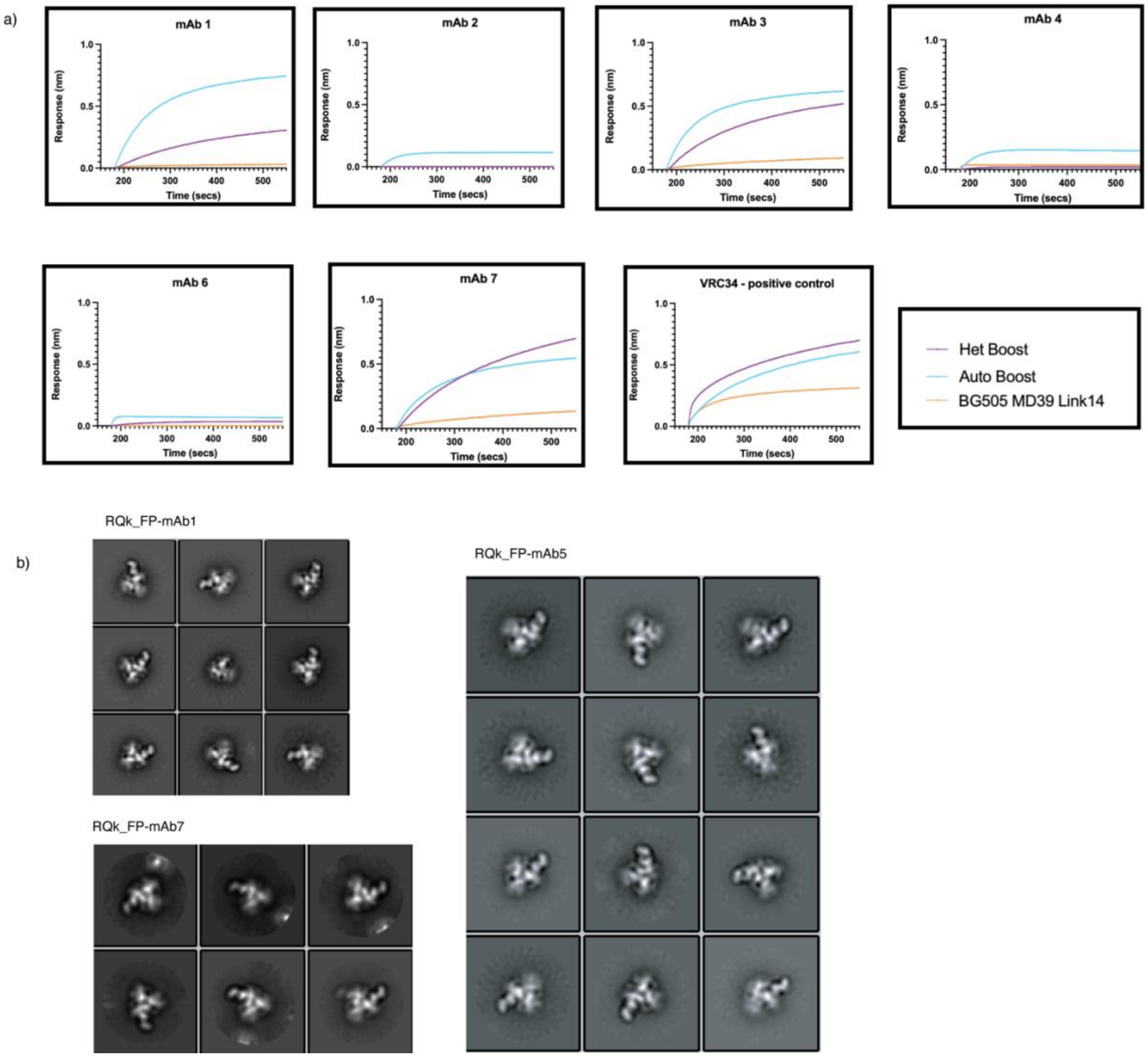

Fig S8. SFS RQk_FP mAb BLI Binding Analysis and nsEM showcase FP and trimer interacting antibodies

a) BLI Binding Analysis for recombinant expressed RQk_FP_mAbs and VRC34 (positive control) to the Heterologous Boost (purple), Autologous Boost (blue) and BG505 MD39 Link14 that does not present FP in a native confirmation due to a linked between C-terminus of gp120 and N-terminus of gp41.

b)) nsEM 2D Classes of RQk_FP_mAbs for mAbs 01, 05, and 07

Table S1. Cross-linked nsEMPEM

Animal ID (Group)	Time point (wk)	Probing Immunogen	2D Classes
LJ66 (1)	14	AMC016	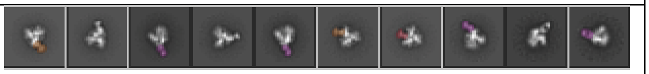
RQk18 (1)	14	AMC016	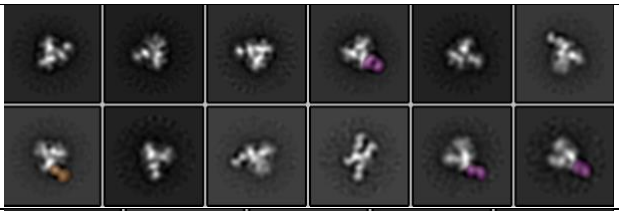
RJy18 (1)	14	AMC016	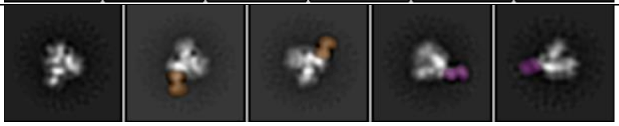
RNp18 (1)	14	AMC016	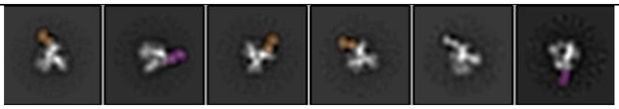

Table S2. FP-sensitive pseudovirus neutralization data weeks 14, 28, 41 and 43.

Study Group#	Animal ID	Week	25710 - 2.43	3988 .25	0077.V 1. C16	CNE19	CNE 56	KER2008 .vrc12	Q2 3.1 7	286.36	BL01. DG
Control	RFv17	14	<20	<20	<20	<20	<20	<20	<20	<20	<20
Control	RTh18	14	<20	<20	<20	<20	<20	<20	<20	<20	<20
Control	RMl18	14	<20	**70**	<20	<20	<20	<20	<20	<20	<20
Control	RMu18	14	**24**	**26**	**22**	<20	<20	<20	<20	<20	<20
Control	RYy18	14	<20	**43**	<20	<20	<20	<20	<20	<20	<20
Control	RUu18	14	<20	<20	<20	<20	<20	<20	<20	<20	**24**
Experimental	LJ66	14	<20	**34**	<20	<20	<20	<20	**25**	<20	**21**
Experimental	RNp18	14	<20	**30**	<20	**25**	<20	<20	<20	<20	<20
Experimental	RNz18	14	<20	<20	<20	<20	<20	<20	<20	<20	<20
Experimental	RJy18	14	<20	**26**	**22**	<20	<20	<20	<20	<20	**26**
Experimental	RTs18	14	<20	**24**	<20	<20	<20	<20	<20	<20	<20
Experimental	RQk18	14	<20	**26**	<20	<20	<20	<20	<20	<20	<20
Control	RFv17	28	<20	<20	<20	<20	<20	<20	<20	<20	<20
Control	RTh18	28	**21**	<20	<20	<20	<20	<20	<20	<20	<20
Control	RMl18	28	<20	<20	<20	<20	<20	<20	<20	<20	<20
Control	RMu18	28	<20	**25**	<20	<20	<20	<20	<20	<20	<20
Control	RYy18	28	<20	<20	<20	<20	<20	<20	<20	<20	<20
Control	RUu18	28	<20	<20	<20	<20	<20	<20	<20	<20	<20
Experimental	LJ66	28	<20	**24**	<20	**38**	<20	<20	<20	<20	<20
Experimental	RNp18	28	<20	**37**	<20	<20	<20	<20	<20	<20	<20
Experimental	RNz18	28	<20	<20	<20	<20	<20	<20	<20	<20	<20
Experimental	RJy18	28	<20	**21**	<20	<20	<20	<20	<20	<20	<20
Experimental	RTs18	28	<20	**38**	<20	<20	<20	<20	<20	<20	<20
Experimental	RQk18	28	**82**	**99**	<20	**289**	<20	**60**	**40**	<20	**123**
Control	RFv17	41	<20	<20	<20	<20	<20	<20	<20	<20	<20
Control	RTh18	41	**78**	<20	<20	**31**	**162**	**34**	**54**	<20	**63**
Control	RMl18	41	<20	<20	<20	<20	<20	<20	<20	<20	<20
Control	RMu18	41	<20	<20	<20	<20	<20	<20	<20	<20	<20
Control	RYy18	41	<20	<20	<20	<20	<20	<20	<20	<20	<20
Control	RUu18	41	<20	**33**	<20	<20	<20	<20	<20	<20	<20
Experimental	LJ66	41	**38**	**32**	<20	**33**	**84**	**28**	**94**	<20	**45**
Experimental	RNp18	41	**23**	**32**	<20	<20	<20	<20	<20	<20	**26**
Experimental	RNz18	41	<20	**51**	<20	<20	<20	<20	<20	<20	<20
Experimental	RJy18	41	<20	**21**	<20	<20	<20	<20	<20	<20	<20
Experimental	RTs18	41	**25**	**52**	<20	**21**	<20	<20	**27**	<20	**25**

Experimental	RQk18	41	**46**	**177**	<20	**754**	<20	**29**	**37**	**22**	**34**
Control	RFv17	43	<20	<20	<20	<20	<20	<20	<20	<20	<20
Control	RTh18	43	<20	<20	<20	<20	<20	<20	<20	<20	**38**
Control	RMl18	43	<20	<20	<20	<20	<20	<20	<20	<20	<20
Control	RMu18	43	<20	<20	<20	<20	<20	<20	<20	<20	<20
Control	RYy18	43	<20	<20	<20	<20	<20	<20	<20	<20	<20
Control	RUu18	43	<20	**36**	<20	<20	<20	<20	<20	<20	<20
Experimental	LJ66	43	<20	**32**	<20	<20	<20	<20	**33**	<20	**36**
Experimental	RNp18	43	<20	**27**	<20	<20	<20	<20	<20	<20	**20**
Experimental	RNz18	43	<20	**46**	<20	<20	<20	<20	<20	<20	<20
Experimental	RJy18	43	<20	**23**	<20	<20	<20	<20	<20	<20	<20
Experimental	RTs18	43	<20	**40**	<20	**22**	<20	<20	**22**	<20	<20
Experimental	RQk18	43	**36**	**149**	<20	**747**	<20	<20	<20	<20	**37**

Values are the serum dilution when relative luminescence units (RLUs) were reduced 50% when compared against virus control wells.

Table S3. BG505 pseudovirus neutralization data weeks 14, 28, 41 and 43.

Study Group	Animal ID	Week	SVA-MLV	BG505	BG505/T332N	BG505/T332N.N611A	BG505/T332N.T465N	BG505/T332N.133aN+136aA	BG505/T332N.S241N.P291T
Control	RFv17	14	<20	<20	<20	<20	<20	<20	<20
Control	RTh18	14	<20	<20	<20	<20	<20	<20	<20
Control	RMl18	14	<20	<20	<20	<20	<20	<20	<20
Control	RMu18	14	<20	<20	<20	<20	<20	<20	<20
Control	RYy18	14	<20	<20	<20	**34**	<20	<20	<20
Control	RUu18	14	<20	<20	<20	<20	<20	<20	<20
Experimental	LJ66	14	<20	<20	<20	<20	<20	<20	<20
Experimental	RNp18	14	<20	<20	<20	<20	<20	<20	<20
Experimental	RNz18	14	<20	<20	<20	<20	<20	<20	<20
Experimental	RJy18	14	<20	<20	<20	**43**	<20	<20	<20
Experimental	RTs18	14	<20	<20	<20	<20	<20	<20	<20
Experimental	RQk18	14	<20	<20	<20	<20	<20	<20	<20
Control	RFv17	28	<20	<20	<20		<20	<20	<20
Control	RTh18	28	<20	<20	<20		<20	<20	<20
Control	RMl18	28	<20	<20	<20		<20	<20	<20
Control	RMu18	28	<20	<20	<20		<20	<20	<20
Control	RYy18	28	<20	<20	<20		<20	<20	<20
Control	RUu18	28	<20	<20	<20		<20	<20	<20
Experimental	LJ66	28	<20	**24**	**24**	**68**	**25**	<20	<20
Experimental	RNp18	28	<20	<20	<20	**167**	<20	<20	<20
Experimental	RNz18	28	<20	<20	<20	<20	<20	<20	<20
Experimental	RJy18	28	<20	<20	<20	**21**	<20	<20	<20
Experimental	RTs18	28	<20	<20	<20	**110**	<20	<20	<20
Experimental	RQk18	28	<20	**56**	**104**	**2160**	**88**	**36**	<20
Control	RFv17	41	<20	<20	<20	**134**	<20	<20	<20
Control	RTh18	41	<20	**37**	**83**	**42**	**90**	<20	**61**
Control	RMl18	41	<20	<20	<20	<20	<20	<20	<20
Control	RMu18	41	<20	<20	<20	<20	<20	<20	<20
Control	RYy18	41	<20	<20	<20	**56**	<20	<20	<20
Control	RUu18	41	<20	<20	<20	**25**	<20	<20	<20
Experimental	LJ66	41	<20	**31**	**57**	**70**	**53**	**30**	**33**
Experimental	RNp18	41	<20	<20	<20	**77**	<20	<20	<20
Experimental	RNz18	41	<20	<20	<20	**39**	<20	<20	<20
Experimental	RJy18	41	<20	<20	<20	**226**	<20	<20	<20

Experimental	RTs18	41	<20	**27**	**27**	**1,320**	**35**	**34**	<20
Experimental	RQk18	41	<20	**221**	**189**	**8,766**	**174**	**178**	**34**
Control	RFv17	43	<20	<20	<20	**49**	<20	<20	<20
Control	RTh18	43	<20	**24**	**44**	**28**	**74**	**24**	**68**
Control	RMl18	43	<20	<20	<20	<20	<20	<20	<20
Control	RMu18	43	<20	<20	<20	<20	<20	<20	<20
Control	RYy18	43	<20	<20	<20	**47**	<20	<20	<20
Control	RUu18	43	<20	<20	<20	<20	<20	<20	<20
Experimental	LJ66	43	<20	**21**	**39**	**49**	**40**	<20	<20
Experimental	RNp18	43	<20	<20	<20	**54**	<20	<20	<20
Experimental	RNz18	43	<20	<20	<20	**66**	<20	<20	<20
Experimental	RJy18	43	<20	<20	<20	**97**	<20	<20	<20
Experimental	RTs18	43	<20	<20	<20	**413**	**26**	<20	<20
Experimental	RQk18	43	<20	**156**	**112**	**5,928**	**110**	**87**	<20

Values are the serum dilution when relative luminescence units (RLUs) were reduced 50% when compared against virus control wells.

Table S4. CH505 and AMC016 pseudovirus neutralization data weeks 14, 28, 41 and 43.

**Study Group#**	**Animal ID**	**Week**	**CH505TF**	**CH505.w4.3**	**AMC016**
Control	RFv17	14	**97**	**2,434**	NA
Control	RTh18	14	<20	**49**	NA
Control	RMl18	14	**41**	**492**	NA
Control	RMu18	14	**195**	**931**	NA
Control	RYy18	14	**370**	**9,200**	NA
Control	RUu18	14	**135**	**1,551**	NA
Experimental	LJ66	14	**253**	**5,636**	NA
Experimental	RNp18	14	**39**	**1,027**	NA
Experimental	RNz18	14	**137**	**9,286**	NA
Experimental	RJy18	14	**89**	**2,086**	NA
Experimental	RTs18	14	**40**	**1,027**	NA
Experimental	RQk18	14	**38**	**795**	NA
Control	RFv17	28	**408**	**19,513**	<20
Control	RTh18	28	<20	**765**	<20
Control	RMl18	28	**199**	**1,095**	<20
Control	RMu18	28	**193**	**1,505**	<20
Control	RYy18	28	**162**	**19,463**	<20
Control	RUu18	28	**238**	**7,975**	<20
Experimental	LJ66	28	**128**	**9,264**	<20
Experimental	RNp18	28	<20	**604**	<20
Experimental	RNz18	28	**36**	**1,973**	<20
Experimental	RJy18	28	**45**	**924**	<20
Experimental	RTs18	28	**21**	**606**	<20
Experimental	RQk18	28	**63**	**1,406**	<20
Control	RFv17	41	**59**	**8,302**	<20
Control	RTh18	41	<20	**1,028**	<20
Control	RMl18	41	**32**	**1,278**	<20
Control	RMu18	41	**59**	**3,987**	<20
Control	RYy18	41	**30**	**10,256**	<20
Control	RUu18	41	**72**	**4,679**	<20
Experimental	LJ66	41	**139**	**28,893**	<20
Experimental	RNp18	41	<20	**2,607**	**21**
Experimental	RNz18	41	**21**	**5,578**	<20
Experimental	RJy18	41	**31**	**1,133**	<20
Experimental	RTs18	41	**27**	**2,559**	<20
Experimental	RQk18	41	**25**	**4,312**	<20

Control	RFv17	43	**32**	**4,342**	<20
Control	RTh18	43	**27**	**1,641**	<20
Control	RMl18	43	<20	**787**	<20
Control	RMu18	43	**37**	**1,995**	<20
Control	RYy18	43	**30**	**8,230**	<20
Control	RUu18	43	**39**	**3,110**	<20
Experimental	LJ66	43	**116**	**18,270**	<20
Experimental	RNp18	43	<20	**1,221**	<20
Experimental	RNz18	43	**21**	**9,555**	<20
Experimental	RJy18	43	**22**	**696**	<20
Experimental	RTs18	43	<20	**771**	<20
Experimental	RQk18	43	**21**	**3,320**	<20

Values are the serum dilution when relative luminescence units (RLUs) were reduced 50% when compared against virus control wells.

Table S5. FP Competition Neutralization Assay

Study Group#	Animal ID	Week	FP-10 Presence (+/-)	Virus	Dilution	% Neutralization
Experimental	LJ66	26	-	CH505TF	43740	0
Experimental	LJ66	26	-	CH505TF	14580	0
Experimental	LJ66	26	-	CH505TF	4860	0
Experimental	LJ66	26	-	CH505TF	1620	0
Experimental	LJ66	26	-	CH505TF	540	0.15709717035
Experimental	LJ66	26	-	CH505TF	180	0.46214242643
Experimental	LJ66	26	-	CH505TF	60	0.75534501646
Control	RFv17	26	-	CH505TF	43740	0
Control	RFv17	26	-	CH505TF	14580	0
Control	RFv17	26	-	CH505TF	4860	0
Control	RFv17	26	-	CH505TF	1620	0.21892452478
Control	RFv17	26	-	CH505TF	540	0.51192253901
Control	RFv17	26	-	CH505TF	180	0.79519183717
Control	RFv17	26	-	CH505TF	60	0.94285010718
Control	RMl18	26	-	CH505TF	43740	0
Control	RMl18	26	-	CH505TF	14580	0
Control	RMl18	26	-	CH505TF	4860	0
Control	RMl18	26	-	CH505TF	1620	0.01366680031
Control	RMl18	26	-	CH505TF	540	0.33030468535
Control	RMl18	26	-	CH505TF	180	0.72027163120
Control	RMl18	26	-	CH505TF	60	0.95726133611
Control	RMu18	26	-	CH505TF	43740	0
Control	RMu18	26	-	CH505TF	14580	0
Control	RMu18	26	-	CH505TF	4860	0
Control	RMu18	26	-	CH505TF	1620	0.02980555827
Control	RMu18	26	-	CH505TF	540	0.29166258883
Control	RMu18	26	-	CH505TF	180	0.65032943650
Control	RMu18	26	-	CH505TF	60	0.87565832052
Control	RYy18	26	-	CH505TF	43740	0.03707936467
Control	RYy18	26	-	CH505TF	14580	0
Control	RYy18	26	-	CH505TF	4860	0.00593838100
Control	RYy18	26	-	CH505TF	1620	0.18755623466
Control	RYy18	26	-	CH505TF	540	0.45850552323
Control	RYy18	26	-	CH505TF	180	0.72470410698

Control	RYy18	26	-	CH505TF	60	0.894956638137603
Control	RUu18	26	-	CH505TF	43740	0
Control	RUu18	26	-	CH505TF	14580	0
Control	RUu18	26	-	CH505TF	4860	0.07072071929
Control	RUu18	26	-	CH505TF	1620	0.12550157378
Control	RUu18	26	-	CH505TF	540	0.36599179802
Control	RUu18	26	-	CH505TF	180	0.67096886217
Control	RUu18	26	-	CH505TF	60	0.87556739794
NA	VRC34.01	NA	-	CH505TF	0.002286	0
NA	VRC34.01	NA	-	CH505TF	0.006858	0
NA	VRC34.01	NA	-	CH505TF	0.020576	0.05799155809
NA	VRC34.01	NA	-	CH505TF	0.061728	0.22483449249
NA	VRC34.01	NA	-	CH505TF	0.185185	0.45373208777
NA	VRC34.01	NA	-	CH505TF	0.555555	0.72631798278
NA	VRC34.01	NA	-	CH505TF	1.666666	0.85426878356
Experimental	LJ66	26	+	CH505TF	43740	0.13686689628
Experimental	LJ66	26	+	CH505TF	14580	0
Experimental	LJ66	26	+	CH505TF	4860	0
Experimental	LJ66	26	+	CH505TF	1620	0
Experimental	LJ66	26	+	CH505TF	540	0.16528020255
Experimental	LJ66	26	+	CH505TF	180	0.50828563581
Experimental	LJ66	26	+	CH505TF	60	0.84156235300
Control	RFv17	26	+	CH505TF	43740	0.14323147689
Control	RFv17	26	+	CH505TF	14580	0
Control	RFv17	26	+	CH505TF	4860	0.01343949386
Control	RFv17	26	+	CH505TF	1620	0.15641525099
Control	RFv17	26	+	CH505TF	540	0.51555944221
Control	RFv17	26	+	CH505TF	180	0.79844231940
Control	RFv17	26	+	CH505TF	60	0.95876155869
Control	RMl18	26	+	CH505TF	43740	0.06162846129
Control	RMl18	26	+	CH505TF	14580	0
Control	RMl18	26	+	CH505TF	4860	0
Control	RMl18	26	+	CH505TF	1620	0
Control	RMl18	26	+	CH505TF	540	0.24551937945
Control	RMl18	26	+	CH505TF	180	0.69031264107
Control	RMl18	26	+	CH505TF	60	0.93484892013
Control	RMu18	26	+	CH505TF	43740	0.10754436422
Control	RMu18	26	+	CH505TF	14580	0

Control	RMu18	26	+	CH505TF	4860	0
Control	RMu18	26	+	CH505TF	1620	0.03276054212
Control	RMu18	26	+	CH505TF	540	0.36940139477
Control	RMu18	26	+	CH505TF	180	0.68810776851
Control	RMu18	26	+	CH505TF	60	0.92155149280
Control	RYy18	26	+	CH505TF	43740	0.15391488004
Control	RYy18	26	+	CH505TF	14580	0.03594283242
Control	RYy18	26	+	CH505TF	4860	0.10322554166
Control	RYy18	26	+	CH505TF	1620	0.24324631495
Control	RYy18	26	+	CH505TF	540	0.50623987775
Control	RYy18	26	+	CH505TF	180	0.77641632439
Control	RYy18	26	+	CH505TF	60	0.93846309269
Control	RUu18	26	+	CH505TF	43740	0.15596063809
Control	RUu18	26	+	CH505TF	14580	0.19096583142
Control	RUu18	26	+	CH505TF	4860	0.24688321815
Control	RUu18	26	+	CH505TF	1620	0.32280357249
Control	RUu18	26	+	CH505TF	540	0.52101479701
Control	RUu18	26	+	CH505TF	180	0.80817102810
Control	RUu18	26	+	CH505TF	60	0.95742272531
NA	VRC34.01	NA	+	CH505TF	0.002286	0.24597399235
NA	VRC34.01	NA	+	CH505TF	0.006858	0.15186912199
NA	VRC34.01	NA	+	CH505TF	0.020576	0.11163588032
NA	VRC34.01	NA	+	CH505TF	0.061728	0.10436207391
NA	VRC34.01	NA	+	CH505TF	0.185185	0.02707788087
NA	VRC34.01	NA	+	CH505TF	0.555555	0.16050676710
NA	VRC34.01	NA	+	CH505TF	1.666666	0.57436361591
Experimental	RJy18	41	-	BG505/T332N.N611A	43740	0.09611230738
Experimental	RJy18	41	-	BG505/T332N.N611A	14580	0
Experimental	RJy18	41	-	BG505/T332N.N611A	4860	0
Experimental	RJy18	41	-	BG505/T332N.N611A	1620	0.17822650268
Experimental	RJy18	41	-	BG505/T332N.N611A	540	0.42299694641
Experimental	RJy18	41	-	BG505/T332N.N611A	180	0.58746720504
Experimental	RJy18	41	-	BG505/T332N.N611A	60	0.74093246843
Experimental	RTs18	41	-	BG505/T332N.N611A	43740	0.02790552366
Experimental	RTs18	41	-	BG505/T332N.N611A	14580	0
Experimental	RTs18	41	-	BG505/T332N.N611A	4860	0.04072452911
Experimental	RTs18	41	-	BG505/T332N.N611A	1620	0.23869350953
Experimental	RTs18	41	-	BG505/T332N.N611A	540	0.50087845123
Experimental	RTs18	41	-	BG505/T332N.N611A	180	0.72242956433

Experimental	RTs18	41	-	BG505/T332N.N611A	60	0.80901831814
Experimental	RQk18	41	-	BG505/T332N.N611A	43740	0.39397278312
Experimental	RQk18	41	-	BG505/T332N.N611A	14580	0.48418955734
Experimental	RQk18	41	-	BG505/T332N.N611A	4860	0.71638286365
Experimental	RQk18	41	-	BG505/T332N.N611A	1620	0.85203454681
Experimental	RQk18	41	-	BG505/T332N.N611A	540	0.93240729232
Experimental	RQk18	41	-	BG505/T332N.N611A	180	0.97955099888
Experimental	RQk18	41	-	BG505/T332N.N611A	60	0.99319356496
Control	RFv17	41	-	BG505/T332N.N611A	43740	0.01472371616
Control	RFv17	41	-	BG505/T332N.N611A	14580	0
Control	RFv17	41	-	BG505/T332N.N611A	4860	0
Control	RFv17	41	-	BG505/T332N.N611A	1620	0
Control	RFv17	41	-	BG505/T332N.N611A	540	0
Control	RFv17	41	-	BG505/T332N.N611A	180	0.28343909460
Control	RFv17	41	-	BG505/T332N.N611A	60	0.69727528948
NA	VRC34.01	NA	-	BG505/T332N.N611A	0.002286	0.50075751722
NA	VRC34.01	NA	-	BG505/T332N.N611A	0.006858	0.56170826012
NA	VRC34.01	NA	-	BG505/T332N.N611A	0.020576	0.79189406180
NA	VRC34.01	NA	-	BG505/T332N.N611A	0.061728	0.88315086854
NA	VRC34.01	NA	-	BG505/T332N.N611A	0.185185	0.93056909531
NA	VRC34.01	NA	-	BG505/T332N.N611A	0.555555	0.93800653715
NA	VRC34.01	NA	-	BG505/T332N.N611A	1.666666	0.96547065166
Experimental	RJy18	41	+	BG505/T332N.N611A	43740	0.30919803952
Experimental	RJy18	41	+	BG505/T332N.N611A	14580	0.04108733115
Experimental	RJy18	41	+	BG505/T332N.N611A	4860	0.01847267059
Experimental	RJy18	41	+	BG505/T332N.N611A	1620	0.08873533255
Experimental	RJy18	41	+	BG505/T332N.N611A	540	0.28573684086
Experimental	RJy18	41	+	BG505/T332N.N611A	180	0.61661230234
Experimental	RJy18	41	+	BG505/T332N.N611A	60	0.84615715375
Experimental	RTs18	41	+	BG505/T332N.N611A	43740	0.30919803952
Experimental	RTs18	41	+	BG505/T332N.N611A	14580	0.02790552366
Experimental	RTs18	41	+	BG505/T332N.N611A	4860	0.07676286519
Experimental	RTs18	41	+	BG505/T332N.N611A	1620	0.15077448157
Experimental	RTs18	41	+	BG505/T332N.N611A	540	0.37534894501
Experimental	RTs18	41	+	BG505/T332N.N611A	180	0.81656460059
Experimental	RTs18	41	+	BG505/T332N.N611A	60	0.95829926465
Experimental	RQk18	41	+	BG505/T332N.N611A	43740	0.70621231309
Experimental	RQk18	41	+	BG505/T332N.N611A	14580	0.56920616897
Experimental	RQk18	41	+	BG505/T332N.N611A	4860	0.70828028473

Experimental	RQk18	41	+	BG505/T332N.N611A	1620	0.84037650789
Experimental	RQk18	41	+	BG505/T332N.N611A	540	0.94124756872
Experimental	RQk18	41	+	BG505/T332N.N611A	180	0.98453831760
Experimental	RQk18	41	+	BG505/T332N.N611A	60	0.99752058397
Control	RFv17	41	+	BG505/T332N.N611A	43740	0.31536567422
Control	RFv17	41	+	BG505/T332N.N611A	14580	0.01677959440
Control	RFv17	41	+	BG505/T332N.N611A	4860	0
Control	RFv17	41	+	BG505/T332N.N611A	1620	0
Control	RFv17	41	+	BG505/T332N.N611A	540	0
Control	RFv17	41	+	BG505/T332N.N611A	180	0.33846407084
Control	RFv17	41	+	BG505/T332N.N611A	60	0.65482745067
NA	VRC34.01	NA	+	BG505/T332N.N611A	0.002286	0.53159569071
NA	VRC34.01	NA	+	BG505/T332N.N611A	0.006858	0.43908117023
NA	VRC34.01	NA	+	BG505/T332N.N611A	0.020576	0.57900182408
NA	VRC34.01	NA	+	BG505/T332N.N611A	0.061728	0.57972742817
NA	VRC34.01	NA	+	BG505/T332N.N611A	0.185185	0.57428539755
NA	VRC34.01	NA	+	BG505/T332N.N611A	0.555555	0.69079322635
NA	VRC34.01	NA	+	BG505/T332N.N611A	1.666666	0.76242244266

Table S6. CryoEM data collection, processing and model building statistics.

Map	Autologous Boost RUu18 wk 14 Polyclonal Fab FP1	Autologous Boost RUu18 wk 14 Polyclonal Fab V1V2V3-1	Autologous Boost RUu18 wk 14 Polyclonal Fab V1V2V3-2	Autologous Boost RUu18 wk 14 Polyclonal Fab V1V2V3-5
EMDB	EMD-49415	EMD-49416	EMD-49417	EMD-49418
**Data collection**				
Microscope	TFS Talos Arctica	TFS Talos Arctica	TFS Talos Arctica	TFS Talos Arctica
Voltage (kV)	200	200	200	200
Detector	Gatan K2 Summit	Gatan K2 Summit	Gatan K2 Summit	Gatan K2 Summit
Recording mode	Counting	Counting	Counting	Counting
Nominal magnification	36,000x	36,000x	36,000x	36,000x
Movie micrograph pixelsize (Å)	1.15	1.15	1.15	1.15
Total dose (e^-/Å^2)	49.92	49.92	49.92	49.92
Defocus range (µm)	-1.8 to -0.8	-1.8 to -0.8	-1.8 to -0.8	-1.8 to -0.8
**EM data processing**				
Number of movie micrographs	7500	7500	7500	7500
Number of molecular projection images in map	143,186	47,505	87,728	171,960
Symmetry	C1	C1	C1	C1
Map pixel size	1.15	1.15	1.15	1.15
Map resolution (FSC 0.143; Å)	3.7	4.0	3.9	3.8
Map sharpening B-factor (Å^2)	-120.2	-112.7	-128.5	-125.5
**Structure building and validation**				
Model Composition				
Non-hydrogen atoms	15017	14897	15064	14784
Protein residues	1873	1856	1880	1851
ligands	64	67	65	60
MolProbity score	1.00	1.05	1.13	0.92
Clashscore	1.05	1.09	1.28	0.69
EMRinger score	2.88	2.21	2.02	2.10
d FSC model (0.5; Å)	3.9	4.2	4.1	4.0
RMSD from ideal				
Bond length (Å)	0.021	0.021	0.021	0.021
Bond angles (˚)	1.712	1.707	1.722	1.717
Rama Outliers (%)	0.00	0.00	0.00	0.00
Side chain rotamer outliers (%)	0.00	0.00	0.00	0.27
Cβ outliers (%)	0.00	0.00	0.00	0.00
PDB	9NHL	9NHM	9NHN	9NHO

Table S6 continued

Map	Autologous Boost RUu18 wk 14 Polyclonal Fab Base 1	Autologous Boost RUu18 wk 14 Polyclonal Fab Base 4	Heterologous Boost RQk18 wk 43 Polyclonal Fab Base A	Heterologous Boost RQk18 wk 43 Polyclonal Fab Base C
EMDB	EMD-49457	EMD-49414	EMD-49411	EMD-49412
**Data collection**				
Microscope	TFS Talos Arctica	TFS Talos Arctica	TFS Titan Krios	TFS Titan Krios
Voltage (kV)	200	200	300	300
Detector	Gatan K2 Summit	Gatan K2 Summit	Gatan K2 Summit	Gatan K2 Summit
Recording mode	Counting	Counting	Counting	Counting
Nominal magnification	36,000x	36,000x	130,000x	130,000x
Movie micrograph pixelsize (Å)	1.15	1.15	1.045	1.045
Total dose (e^-/Å^2)	49.92	49.92	50.29	50.29
Defocus range (µm)	-1.8 to -0.8	-1.8 to -0.8	-1.8 to -0.8	-1.8 to -0.8
**EM data processing**				
Number of movie micrographs	7500	7500	11745	11745
Number of molecular projection images in map	181,253	110,868	357902	145,407
Symmetry	C1	C1	C1	C1
Map pixel size	1.15	1.15	1.045	1.045
Map resolution (FSC 0.143; Å)	3.8	3.89	3.0	3.1
Map sharpening B-factor (Å^2)	-124.0	-116.9	-94.97	-89.0
**Structure building and validation**				
Number of atoms in deposited model				
Non-hydrogen atoms	14956	14953	15606	15028
Protein residues	1879	1859	1889	1844
ligands	58	69	97	75
MolProbity score	1.11	1.07	0.59	0.90
Clashscore	1.39	0.88	0.23	0.88
EMRinger score	2.61	2.42	4.40	4.05
d FSC model (0.5; Å)	3.9	4.0	3.1	3.2
RMSD from ideal				
Bond length (Å)	0.021	0.021	0.005	0.005
Bond angles (˚)	1.706	1.731	0.935	0.941
Rama Outliers (%)	0.00	0.00	0.00	0.00
Side chain rotamer outliers (%)	0.00	0.14	0.00	0.00
Cβ outliers (%)	0.00	0.00	0.00	0.00
PDB	9NI9	9NHK	9NHH	9NHI

Table S6 continued

Map	Heterologous Boost RQk18 wk 43 Polyclonal Fab FP-A	Heterologous Boost RQk_FP_mAb_05
EMDB	EMD-49413	
**Data collection**		
Microscope	TFS Titan Krios	TFS Glacios
Voltage (kV)	300	200
Detector	Gatan K2 Summit	TFS Falcon 4
Recording mode	Counting	Counting
Nominal magnification	130,000x	190,000x
Movie micrograph pixelsize (Å)	1.045	0.725
Total dose (e^-/Å^2)	50.29	45
Defocus range (µm)	-1.8 to -0.8	-2.0 to -1.0
**EM data processing**		
Number of movie micrographs	11,745	16,477
Number of molecular projection images in map	254,630	111,683
Symmetry	C1	C1
Map pixel size	1.045	0.725
Map resolution (FSC 0.143; Å)	3.0	3.9
Map sharpening B-factor (Å^2)	-90.3	-93.5
**Structure building and validation**		
Number of atoms in deposited model		*NA*
Non-hydrogen atoms	15437	
Protein residues	1905	
ligands	76	
MolProbity score	0.94	
Clashscore	0.85	
EMRinger score	3.42	
d FSC model (0.5; Å)	3.2	
RMSD from ideal		
Bond length (Å)	0.005	
Bond angles (˚)	0.973	
Rama Outliers (%)	0.00	
Side chain rotamer outliers (%)	0.00	
Cβ outliers (%)	0.00	
PDB	9NHJ	

Table S7. RQk_FP_mAb Design – Heavy chain

H-chain name	Additional mutations	Top Score Hit #ID	Assigned FWRH1 region	Assigned JH region
RQk_FP1_HC_v1		980571, 979518	IGHV4-117*01	IGHJ4-3*01
RQk_FP1_HC_v2	G59R	980571, 979518	IGHV4-117*01	IGHJ4-3*01
RQk_FP1_HC_v3		980571, 979518	IGHV4-117*01	IGHJ4-3*01
RQk_FP1_HC_v4	D114R	980571, 979518	IGHV4-117*01	IGHJ4-3*01

Table S8. RQk_FP_mAb Design – Light chain

L-chain name	Additional mutations	Top Score Hit #ID
RQk_FP1_LC_v1		flexible CDRL3, ordered (SSSPV)
RQk_FP1_LC_v2	S108N, S114N, V116P	Based on top pick CDRH3s (NSNPP)

Table S9. RQk_FP_mAb Design – Heavy chain and Light Chain Pairing

# Antibody	H-chain	L-chain
RQk_FP1_Ab1_IgG	RQk_FP1_HC_v1	RQk_FP1_LC_v1
RQk_FP1_Ab2_IgG	RQk_FP1_HC_v2	RQk_FP1_LC_v1
RQk_FP1_Ab3_IgG	RQk_FP1_HC_v3	RQk_FP1_LC_v1
RQk_FP1_Ab4_IgG	RQk_FP1_HC_v4	RQk_FP1_LC_v1
RQk_FP1_Ab5_IgG	RQk_FP1_HC_v1	RQk_FP1_LC_v2
RQk_FP1_Ab6_IgG	RQk_FP1_HC_v2	RQk_FP1_LC_v2
RQk_FP1_Ab7_IgG	RQk_FP1_HC_v3	RQk_FP1_LC_v2
RQk_FP1_Ab8_IgG	RQk_FP1_HC_v4	RQk_FP1_LC_v2

Table S10. AMC016 and BG505 pseudovirus neutralization data for RQk_FP_mAbs

mAb	SVA-MLV	BG505	BG505/ T332N	BG505/ T332N. N611A	BG505/ T332N. T465N	BG505/ T332N. 133aN+ 136aA	BG505/ T332N. S241N. P291T	AMC016
RQk_FP_mAb _01	>50.0	>50.0	>50.0	>50.0	>50.0	>50.0	>50.0	>50.0
RQk_FP_mAb _03	>50.0	>50.0	>50.0	>50.0	>50.0	>50.0	>50.0	>50.0
RQk_FP_mAb _05	>50.0	>50.0	>50.0	>50.0	>50.0	>50.0	>50.0	>50.0
RQk_FP_mAb _07	>50.0	>50.0	>50.0	>50.0	>50.0	>50.0	>50.0	>50.0

Table S11. FP-sensitive pseudovirus neutralization data for RQk_FP_mAbs

mAb	3988.25	0077.v1.c16	25710-2.43	286_36	BL01.DG	CNE19	CNE56	KER2008.vrc12	Q23.17
RQk_FP_mAb_01	>50.0	>50.0	>50.0	>50.0	>50.0	>50.0	>50.0	>50.0	>50.0
RQk_FP_mAb_03	>50.0	>50.0	>50.0	>50.0	>50.0	>50.0	>50.0	>50.0	>50.0
RQk_FP_mAb_05	>50.0	>50.0	>50.0	>50.0	>50.0	>50.0	>50.0	>50.0	>50.0
RQk_FP_mAb_07	>50.0	>50.0	>50.0	>50.0	>50.0	>50.0	>50.0	>50.0	>50.0

Table S12: FNA Phenotyping Staining Panel

Viability	e506
CD8a	BV510
PD-1 (Clone EH12.2H7)	BV605
CD4 (Clone OKT-4)	BV785
CD20 (Clone 2H7)	Ax488
IgG (Clone G18-145)	AF700
CXCR5 (Clone Mu5UBEE)	PECy7
IgM (Clone G20-127)	PerCP-Cy5.5
CD71 (Clone L01.1)	PE-CF594
CD38 (Clone OKT)	PE

Table S13: 10X B cell Staining Panel

Live/Dead	APCe780
CD8a	APCe780
CD4 (Clone SK3)	APCe780
CD16 (ebioCD16)	APCe780
CD20 (Clone 2H7)	Ax488
IgG (Clone G18-145)	PECy7
IgM (Clone G20-127)	PerCP-Cy5.5
CD71 (Clone L01.1)	PE-CF594
CD38 (Clone OKT)	PE

Supplementary Data 01
Bulk B cell repertoire heavy chain sequencing analysis of RQk18 PBMC from week 42

Supplementary Data 02
Bulk B cell repertoire light chain sequencing analysis of RQk18 PBMC from week 42

Supplementary Data 03
10X Genomics meta-analysis of RQk18 week 14 PBMC clonotypes.
